# Intussusception of the Ilium in a Two-Year-Old Stallion

**Published:** 1882-07

**Authors:** George M. Parkinson


### II.—INTUSSUSCEPTION OF THE ILIUM IN A TWO-YEAR-OLD STALLION.

REPORTED BY GEORGE M. PARKINSON, D.V.S.

*Previous History:* About one year ago the colt passed two or three worms which resembled angle worms in shape and length, but lighter in color (probably lumbricoid). April 8th, first saw animal. During the previous two or three days the groom noticed the animal turning his head and looking at the right flank, would lie down and roll, then get up, start off and run, but this did not cause any alarm. The colt had been kept on low damp pasture land.

*Examination:* Found the animal thin in flesh, pulse feeble and scarcely perceptible, soft and compressible; number of pulsations per minute, sixty; respirations about normal; temperature 104.5° F.; appeared to be in constant pain, walking around in a circle with extended head and pointing towards the floor.

At short intervals he would lie down on the floor, roll on his back and extend all four limbs, and remain in this position for about one minute; would then get up, look anxiously at right flank, and then sit down upon haunches. These movements were frequently repeated. All the visible mucous membranes were slightly injected. There was slight tympanitis over the right iliac region. There was no attempt to evacuate the bowels. There was profuse sweating, and he soon became pulseless and died after an illness of only twenty-seven hours.

During this time I emptied the bladder by means of a catheter; gave opium in two-ounce doses, and chloral hydrate in one-ounce doses; used enemata of soap-suds medicated with gum-assofœtida, also the following bolus:

℞

Aloes Barb., ℥ vi.

Zingib. Rad. Pulv., ℥ i.

Nuces Vomic. Pulv., gr. xxx.

Nx. et first in bolus, No. i.

Sig. at once.

*Necropsy:* Twenty-four hours after death great quantities of long round worms in the stomach and bowels, also patches of congestion. The colon was markedly inflamed. The last foot of the ilium had slipped through the ileo-coreal valve and become invaginated, and was in a gangrenous state. What fœcal matter there was in the colon was dry and hard, stomach and small intestines filled with fluid.

MIDDLETOWN, CONN.

[The worms in this case, we are inclined to think, were the Strongylus Gigas.—ED.]

